# Clinical Significance of Shared T Cell Epitope Analysis in Early *De Novo* Donor-Specific Anti-HLA Antibody Production After Kidney Transplantation and Comparison With Shared B cell Epitope Analysis

**DOI:** 10.3389/fimmu.2021.621138

**Published:** 2021-03-26

**Authors:** Toshihide Tomosugi, Kenta Iwasaki, Shintaro Sakamoto, Matthias Niemann, Eric Spierings, Isao Nahara, Kenta Futamura, Manabu Okada, Takahisa Hiramitsu, Asami Takeda, Norihiko Goto, Shunji Narumi, Yoshihiko Watarai, Takaaki Kobayashi

**Affiliations:** ^1^ Department of Transplant Surgery, Nagoya Daini Red Cross Hospital, Nagoya, Japan; ^2^ Department of Renal Transplant Surgery, Aichi Medical University School of Medicine, Nagakute, Japan; ^3^ Department of Kidney Diseases and Transplant Immunology, Aichi Medical University School of Medicine, Nagakute, Japan; ^4^ Department of Histocompatibility Laboratory, Nagoya Daini Red Cross Hospital, Nagoya, Japan; ^5^ PIRCHE AG, Berlin, Germany; ^6^ Center of Translational Immunology, UMC Utrecht, Utrecht, Netherlands; ^7^ Department of Pharmacoepidemiology, Kyoto University Graduate School of Medicine, School of Public Health, Kyoto, Japan; ^8^ Department of Nephrology, Nagoya Daini Red Cross Hospital, Nagoya, Japan

**Keywords:** kidney transplantation, T cell epitope analysis, B cell epitope analysis, donor-specific antibody, PIRCHE-II

## Abstract

In pre-sensitizing events, immunological memory is mainly created *via* indirect allorecognition where CD4^+^ T cells recognize foreign peptides in the context of self-HLA class II (pHLA) presented on antigen-presenting cells. This recognition makes it possible for naive CD4^+^ T-helper cells to differentiate into memory cells, resulting in the creation of further antibody memory. These responses contribute to effective secretion of donor-specific anti-HLA antibodies (DSA) after second encounters with the same peptide. Preformed donor-reactive CD4^+^ memory T cells may induce early immune responses after transplantation; however, the tools to evaluate them are limited. This study evaluated shared T cell epitopes (TEs) between the pre-sensitizing and donor HLA using an *in silico* assay, an alternative to estimate donor-reactive CD4^+^ memory T cells before transplantation. In 578 living donor kidney transplants without preformed DSA, 69 patients had anti-HLA antibodies before transplantation. Of them, 40 had shared TEs and were estimated to have donor-reactive CD4^+^ memory T cells. *De novo* DSA formation in the early phase was significantly higher in the shared TE-positive group than in the anti-HLA antibody- and shared TE-negative groups (p=0.001 and p=0.02, respectively). In conclusion, evaluation of shared TEs for estimating preformed donor-reactive CD4^+^ memory T cells may help predict the risk of early *de novo* DSA formation after kidney transplantation.

## Introduction

Adaptive immunity creates immunological memory after the first response to a specific foreign antigen; this memory leads to an enhanced rapid response to subsequent exposure to the same antigen and is important in organ transplantation (1). Immunological memory is created during pre-sensitizing events such as blood transfusion, pregnancy, and prior organ transplantation, and is reflected by the presence of anti-HLA antibodies (2). Production of antibody memory involves the indirect allorecognition pathway in which the T cell receptor of recipient naïve CD4^+^ T-helper cells first recognizes a foreign-HLA-derived peptide in the context of recipient HLA class II (pHLA), which are presented on the recipient antigen-presenting cells (APCs) (3). Recipient naïve B cells, one of the APCs, also presents the foreign-HLA-derived pHLA, while specifically recognizing the foreign HLA with their B cell receptor. Unlike T cell receptors, B cell receptors recognize fragments on the tertiary structure of proteins, which are in structurally close contact (4). This indirect allorecognition with the foreign-HLA-specific T cell receptor allows naive CD4^+^ T-helper cells to differentiate into memory cells, thereby helping naïve B cells with the foreign-HLA-specific B cell receptor to differentiate into antibody-producing plasma cells and memory B cells (5, 6). During this process, memory T cells and B cells memorize the molecular components known as epitope, not the foreign HLA as a whole (4). These memory cells lead an enhanced, rapid response after second encounters with the same epitopes derived from donor HLA (7–9).

The HLA loci have the most polymorphic regions in the human genome, with over 25,000 HLA alleles observed so far (10); however, the different HLA alleles often share epitopes with each other (11). Shared epitopes among multiple HLAs make it possible for memory cells to be recalled by not only the past-sensitizing HLAs, but also by the newly encountering HLAs, which creates a solid immunological defense system toward alloantigens (12). Reactivity toward shared epitopes was first confirmed in anti-HLA antibodies (13), whose recognizing region explained as B cell epitopes (BEs) were shared among multiple HLAs. The HLA groups shared with BEs were historically classified as cross-reactive-antigen groups (CREG) and received considerable attention as a risk predictor after transplantation. For example, organ allocation based on shared BEs, called CREG matching, was shown to reduce the frequency of sensitization to the donor HLA in a multicenter study (14). Furthermore, some recipients with preformed non-donor-specific anti-HLA antibodies (non-DSA) toward donor-CREG were reportedly associated with increased risks of early antibody-mediated rejection after transplantation (15). Recently, BEs in each HLA allele can be easily calculated using an *in silico* analytical tool known as the HLAMatchmaker. The analysis of shared BEs, such as CREG, has progressed to the current practices for solid organ transplantation. However, the pathological mechanisms by which organ allocation or risk stratification of non-DSA based on shared BEs affects the prognosis of transplant recipients remains unclear. These BE analyses could be a marker for structural similarity between each HLA molecule; however, these analyses are insufficient for estimating the mechanisms of acquired immunity because T cell reactivity is not considered in these methods. When focusing on the processes that create immunological memory, a joint approach of analyzing both BE and T cell epitope (TE) might give a comprehensive picture of pre-sensitization.

The reactivity of memory T cells toward shared TEs between the pre-sensitizing HLA and donor HLA may increase the risk of progression to early onset of rejection, resulting in poor graft prognosis (16); however, the tools to detect them are limited. Currently, enzyme-linked immunospot (ELISPOT) assay for the detection of allospecific cytokines produced by individual human peripheral blood lymphocytes is one of the main tools (17–19). Furthermore, detecting donor-HLA-reactive memory CD4^+^ T cells *via* the indirect allorecognition pathway is technically difficult, although this pathway is thought to be a key mechanism in the progression of alloreactivity in organ transplantations (20).

**Figure 1:**
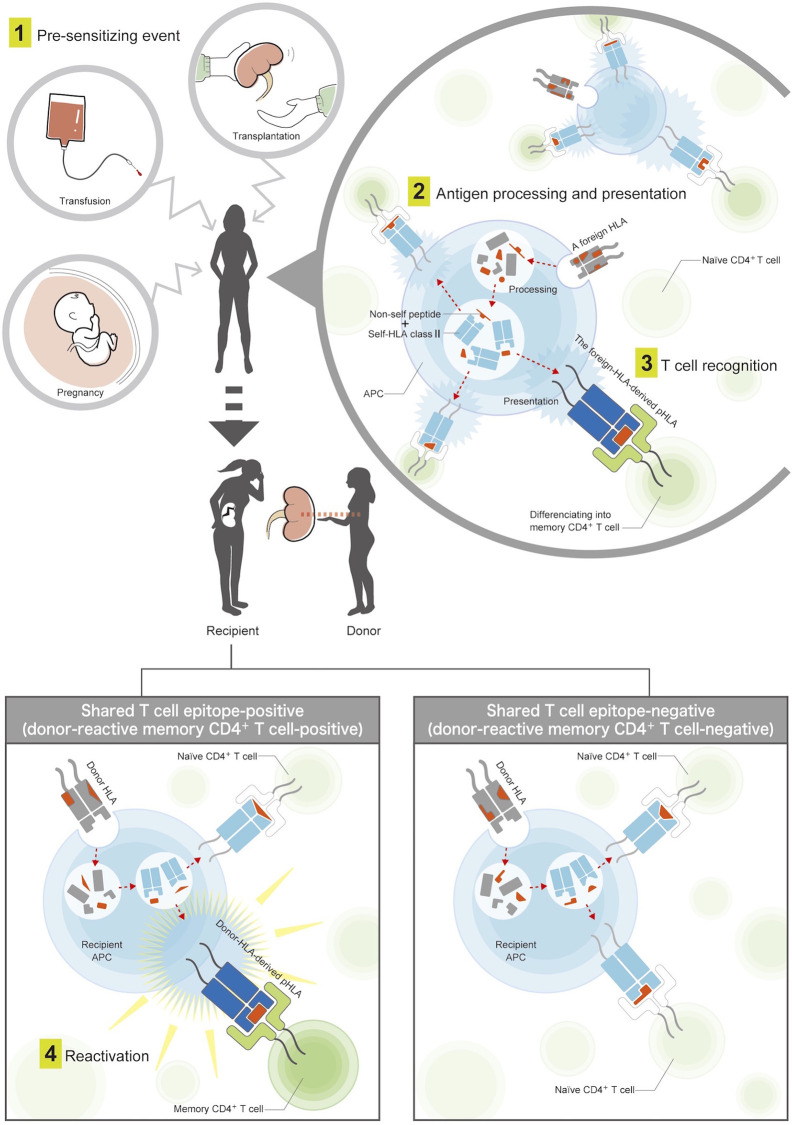
Mechanisms of pre-sensitization *via* the indirect T cell-allorecognition pathway. (1) A patient is first exposed to foreign HLAs by pre-sensitizing events, such as blood transfusion, pregnancy, or prior organ transplantation. (2) The foreign HLAs are processed into smaller peptides by the patient’s antigen-presenting cells. Among them, non-self peptides are loaded onto the recipient HLA class II, and the antigens are presented on the cell surface. (3) Patient’s naïve CD4^+^ T-helper cells recognize T cell epitopes consisting of the foreign-HLA-derived peptide in the context of recipient HLA class II (pHLA), which allows naive CD4^+^ T-helper cells to differentiate into memory cells. (4) These memory CD4^+^ T cells lead to an enhanced rapid response after second encounters with the same pHLAs derived from the donor HLA. The donor-reactive memory CD4^+^ T-helper cells were considered to be positive if the foreign-HLA-derived pHLAs were shared with donor-HLA-derived pHLAs, and negative if not shared.

Therefore, this study used the predicted indirectly recognizable HLA epitopes (PIRCHE)-II algorithm (21), an *in silico* assay focusing on the indirect allorecognition pathway, as an easy and alternative tool to estimate donor-reactive memory CD4^+^ T cells. We hypothesized that the evaluation of shared TEs between the pre-sensitizing HLA and donor HLA for the purpose of estimating preformed donor-reactive memory CD4^+^ T cells may be reasonable and helpful in predicting the risk of early *de novo* DSA (dnDSA) formation after transplantation (**Figure 1**), and we compared the efficacy of the risk predictor with the conventional evaluation of shared BEs.

## Materials and Methods

### Patients

A total of 679 living donor kidney transplants from the Nagoya Daini Red Cross Hospital between 2012 and 2018 were eligible for this retrospective single-center cohort study. All recipients and donors were of Japanese origin. The final follow-up of all analyses was December 31, 2019. Informed consent was obtained from patients and donors in accordance with the Declaration of Helsinki. The study was approved by the Aichi Medical University Institutional Review Board.

### Patient and Donor HLA Typing

Alleles at the HLA-A, -B, -DRB1, and -DQB1 loci were identified in all 679 pairs by xMAP^®^ Technology of Luminex Corp. using PCR-sequence specific oligonucleotide (SSO) probes (WAKFlow HLA Typing kit, Wakunaga Pharmaceutical Co. Ltd., Hiroshima, Japan or One Lambda, Canoga Park, CA, USA) at high resolution. The typing kit can identify alleles with a frequency of 0.1% or more by combining the results with the information based on epidemiological allele frequency in the Japanese population (22). DRB3/4/5 and DQA1 were estimated using a local haplotype frequency dataset of 916 unrelated Japanese individuals (23). This estimation is widely used in the Japanese population because their haplotype frequencies have been concentrated based on a single ethnicity (24).

### HLA Antibody Surveillance and Definition of Pre-Sensitizing HLA

Within the six months prior to transplantation, all patients were tested by complement-dependent cytotoxicity crossmatch (25), flow cytometry cross matches (26), and anti-HLA antibody screening (27, 28) with the use of flow panel reactive antibody (PRA) (One Lambda, California, US). Preformed DSA and non-DSA were determined by the Luminex-based LABScreen single antigen beads (SAB) assay (One Lambda, California, US). All serum samples used for antibody analysis were treated with EDTA to prevent the prozone effect. Mean fluorescence intensity (MFI) values >1000 were regarded as positive for the SAB assay. In patients with preformed non-DSA, the HLA allele with the highest MFI value for each of HLA class I and class II before transplantation was considered as a pre-sensitizing HLA. All characteristics of non-DSA (MFI values >1000), as well as TE and BE counts shared with the donor HLA, are shown in **Supplementary Tables 1** and **2**. Post-transplantation anti-HLA antibody surveillance was annually performed by the flow PRA and SAB assays, according to the manufacturer’s instructions. In cases of impaired allograft function, anti-HLA antibody surveillance was added accordingly. The HLA loci A, B, DRB1, DRB3/4/5, DQB1, and DQA1 were considered for the definition of DSA.

### Shared TE-Analysis as a Tool to Estimate Donor-Reactive Memory CD4+ T-Helper Cells

The HLA-derived pHLAs, namely TEs, were all calculated using the latest version of the PIRCHE-II algorithm version 3.0 (PIRCHE AG, Berlin, Germany). HLA DRB1, DRB3/4/5, and DQB1/DQA1 were taken into consideration as presenting loci, while A, B, DRB1, DRB3/4/5, DQB1, and DQA1 were considered as presented loci. The binding probability of the presented peptide and presenting HLA class II was estimated; affinities with an IC50 of <1000 nM (27) were included in our analysis and the sum total of estimated TE-mismatch in each donor and recipient pair was defined as the PIRCHE-II score. The PIRCHE-II scores in this study were higher in range than those in previously reported (29, 30) in which only DRB1 was considered as the presenting locus. Based upon reports (29, 30), the natural logarithm of the PIRCHE-II scores [ln(PIRCHE-II)] were used to calculate the hazard ratio in the Cox proportional hazards regression model.

In patients with preformed non-DSA, TEs derived from presensitizing HLA werecalculated using PIRCHE-II and compared with calculated TEs derived from the donor HLA. We considered the shared TEs to be positive and estimated the presence of donor reactive memory CD4+ T-helper cells if the two sets of TEs shared at least one pHLA, and negative if no pHLA were shared (**Figure 1**); for each non-DSA, TE counts shared with the donor HLA are shown in **Supplementary Table 1**.

### HLAMatchmaker Analysis

BE mismatch levels for HLA-A, -B, -DRB1, -DRB3/4/5, and -DQB1/DQA1 were determined using the HLAMatchmaker software version 3.0 in each donor and recipient pair. The HLAMatchmaker score was considered as the total number of mismatched eplets, including antibody verified and non-verified eplets. The HLAMatchmaker score of 10 increments was also used to calculate the hazard ratio in the Cox proportional hazards regression model (29).

### Shared BE Analysis

In patients with preformed non-DSA, similarly to the shared-TE analysis, the BEs derived from pre-sensitizing HLA were estimated using the HLAMatchmaker software, and were compared with the calculated BEs derived from the donor HLAs. The shared BEs were determined as positive if the two sets of BEs shared at least one eplet, and negative if no eplets were shared; for each non-DSA, the BE counts shared with the donor HLA are shown in **Supplementary Table 2**.

### Protocol Biopsies and Diagnosis of Rejection

Protocol biopsies were routinely performed on all patients at 2-3 weeks and 12 months after transplantation. In cases with impaired allograft function, biopsies were added accordingly. An experienced pathologist diagnosed antibody-mediated rejection and T cell-mediated rejection according to the revised Banff classification (31–34).

### Immunosuppression

All patients received basiliximab as induction immunosuppression therapy. Patients who received an ABO-incompatible graft were additionally pretreated with rituximab and plasma exchange and/or double-filtration plasmapheresis before transplantation. Maintenance immunosuppression therapy consisted of triple therapy with prednisolone, calcineurin inhibitor (tacrolimus or cyclosporine), and mycophenolic acid. Some patients received the mammalian target of rapamycin inhibitor (everolimus) instead of mycophenolic acid.

### Statistical Analysis

All statistical analyses were conducted using SPSS statistical software version 21 (IBM Corp., Armonk, NY). Continuous variables are expressed as a mean and standard deviation or median and interquartile range (IQR) according to their distribution and analyzed using the Student’s *t*-test or Mann-Whitney *U* test. In cases of comparison across three groups, one-way analysis of variance (ANOVA) with the Kruskal-Wallis test was used. The Tukey honestly significant difference test was performed under the significant result of ANOVA for multiple comparisons. Categorical variables are expressed as a frequency and percentage and were examined using the Fisher exact or Chi-squared test according to the expected count. DSA-free graft survival was defined as the time between kidney transplantation and the date of the last anti-HLA antibody surveillance without DSA detection. Time-dependent outcomes such as DSA-free survival rates were estimated using the Kaplan-Meier survival curves and Breslow tests. The starting time point for these time-dependent survival analyses was determined as the day of transplantation. In the analysis of DSA-free survival, censoring occurred at the time of the last anti-HLA antibody surveillance. The Cox proportional hazards regression model for univariate analysis was used to find variables that affected DSA-free survival. Additionally, multivariate analysis with forced entry model was performed and adjusted for potential confounding factors that were selected based on the previous report (29) to assess the strength of the association after adjustment. P-values less than 0.05 were considered statistically significant. The relationships between the PIRCHE-II and HLAMatchmaker score and between the shared-TE and shared-BE counts were investigated using the Spearman’s rank-correlation coefficient (rho).

## Results

### Patient Background

**Figure 2:**
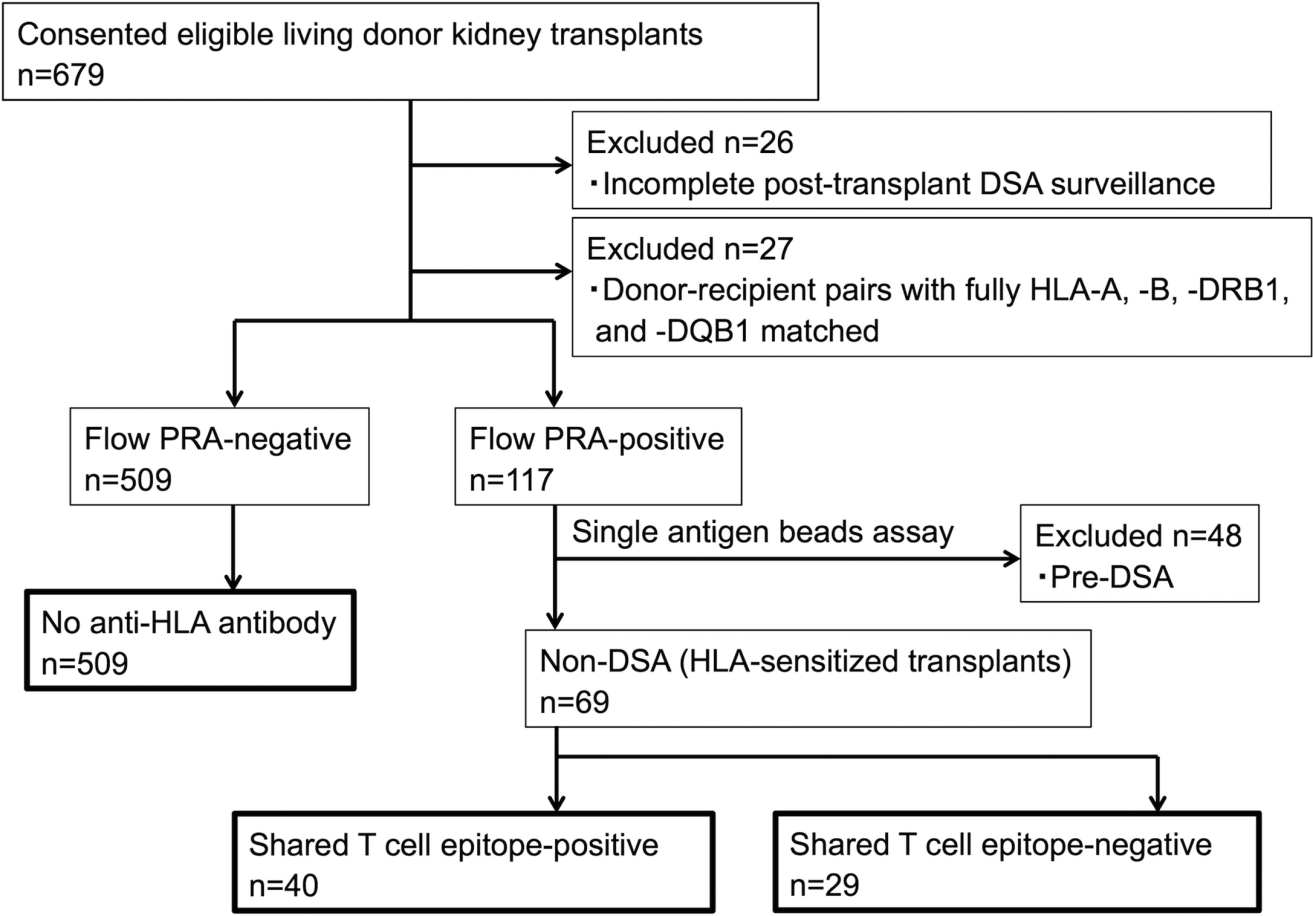
Patient flowchart. Pre-DSA, preformed-donor-specific anti-HLA antibodies; non-DSA, non-donor-specific anti-HLA antibodies; PRA, panel reactive antibody.

Consecutive living donor kidney transplants (n=679) were eligible for this study. We included only kidney transplants with complete HLA typing at high-resolution level (HLA-A, -B, -DRB1, and -DQB1) and pre- and post-transplantation follow-up for dnDSA surveillance. Twenty-six transplants were excluded because of incomplete post-transplantation DSA surveillance, and 27 patients whose HLA (HLA-A, -B, -DRB1, and -DQB1) were fully matched with the donors were excluded from the study (n=27) because dnDSA would not be detected in such patients; furthermore, 48 patients with preformed DSA were excluded. A total of 578 patients remained for analysis and were classified into either the preformed anti-HLA antibody-positive group (n=69) or anti-HLA antibody-negative group (n=509). The 69 HLA-sensitized transplants without preformed DSA were classified into either the shared TE-positive group (n=40) or shared TE-negative group (n=29) (**Figure 2**).

**Table 1** describes the baseline characteristics of the shared TE-positive, shared TE-negative, and no anti-HLA antibody groups. The median follow-up period after transplantation was 47 months (IQR, 29-71.75 months; range, 1-95 months). Sensitizing events were seen more frequently in the HLA pre-sensitized (non-DSA) group, although there were no statistically significant differences (p=0.06). There were also no statistically significant differences in the other background characteristics including baseline immunosuppression therapy at transplantation, TE-mismatch count (calculated by PIRCHE-II), and BE-mismatch count (calculated by the HLAMatchmaker).

**Table 1:** Patient characteristics.

Characteristics	Anti-HLA ab (non-DSA)		No anti-HLA ab n = 509	*P*-value
	Shared TE-positive	Shared TE-negative		
	n = 40	n = 29		
**Donor**				
Age, years, mean (SD)	60.1 (9.3)	60.2 (10.0)	58.1 (10.1)	0.24
Female sex, n (%)	28 (70)	20 (69.0)	336 (66.0)	0.84
Relationship, n (%)				0.65
Unrelated	22 (55)	12 (41.4)	284 (55.8)	
Related (haplotype-unrelated)	2 (5)	2 (6.9)	21 (4.1)	
Related (haplotype-related*)	16 (40)	15 (51.7)	204 (40.1)	
**Recipient**				
Age, years, mean (SD)	49.5 (18.2)	49.0(12.8)	47.6 (16.3)	0.65
Female sex, n (%)	15 (37.5)	13 (44.8)	155 (30.5)	0.19
ABO-i, n (%)	14 (35)	9 (31.0)	184 (36.1)	0.85
**ESRD causes, n (%)**				
Glomerulonephritis	11 (27.5)	11 (37.9)	179 (35.2)	0.58
Polycystic kidney disease	4 (10)	1 (3.4)	33 (6.5)	0.54
Diabetes	10 (25)	5 (17.2)	116 (22.8)	0.74
Other	15 (37.5)	12 (41.4)	181 (35.6)	0.61
**Months on dialysis, n (%)**				0.47
0 (preemptive transplantation)	18 (45)	13 (44.8)	272 (53.4)	
-6	4 (10)	6 (20.7)	66 (13.0)	
6-47	10 (25)	8 (27.6)	108 (21.2)	
48-	8 (20)	2 (6.9)	63 (12.4)	
Pre-sensitizing event**, n (%)	19 (47.5)	14 (48.3)	170 (33.4)	0.06
Pre-transplantation	3 (7.5)	0 (0)	14 (2.8)	0.15
Pregnancy***	10 (66.7)	11 (84.6)	95 (61.3)	0.24
Transfusion	10 (25)	5 (17.2)	96 (18.9)	0.14
**Histocompatibility (HLA-A, B, DRB1/3/4/5, DQB1, DQA1), median (IQR)**				
HLA mismatches	6 (5 - 9)	5 (5 – 9)	6 (5 - 10)	0.34
HLAMatchmaker score				
AB	11 (5 - 16)	10 (4 – 16)	12 (7 - 16)	0.09
DR	8.5 (6 - 18)	7 (4 – 14)	11 (5 - 18)	0.51
DQ	13.5 (5 - 23)	12 (5 – 22)	13 (7 – 22)	0.92
Total	36 (24 – 49.5)	29 (19 – 41)	37 (25 – 51)	0.30
PIRCHE-II score	184.5 (120 – 280.5)	168 (112 - 260)	199 (131 – 298)	0.16
**Baseline immunosuppression at transplant, n (%)**				
Steroid	40 (100)	29 (100)	509 (100)	–
Tacrolimus	24 (60)	22 (75.9)	338 (66.4)	0.39
Cyclosporin	16 (40)	7 (24.1)	171 (33.6)	0.39
Everolimus	8 (20)	7 (24.1)	103 (20.2)	0.88
Micophenolic acid	31 (77.5)	22 (75.9)	400 (78.6)	0.93
**Induction, n (%)**				
Basiliximab	40 (100)	29 (100)	509 (100)	–
Thymoglobulin	0 (0)	0 (0)	0 (0)	–
**Desensitization, n (%)**				
Anti-CD20 therapy	11 (27.5)	7 (24.1)	154 (30.3)	0.74
Plasmapheresis	14 (35)	9 (31.0)	191 (37.5)	0.75
IVIG	0 (0)	0 (0)	2 (0.4)	0.87

*Haplotype-related donors shared one HLA haplotype with the recipients.

**Pre-sensitizing events were recorded as 1 per patient, even if the patient had multiple pre-sensitizing events.

***The percentage was calculated using only the female population.

Ab, antibody; non-DSA, non-donor-specific anti-HLA antibodies; TE, T cell epitope; SD, standard deviation; ABO-I, ABO-incompatible transplantation; ESRD, end stage renal disease; PIRCHE, predicted indirectly recognizable HLA epitopes; IVIG, intravenous immunoglobulin; HLA, human leukocyte antigen; IQR, interquartile range.

### Characteristics of Estimated Pre-Sensitizing HLA

There were 69 HLA pre-sensitized (non-DSA) patients. Of these patients, 44 (63.8%) had only HLA class I, 15 (21.7%) had only HLA class II, and 10 (14.5%) had both HLA class I and II non-DSA. The median highest MFI of the preformed non-DSA before transplantation was 2,436.5 (IQR 1,462.75-6,134). Thirty-seven (53.6%) patients showed low-level MFI <3,000, 21 (30.4%) showed moderate-level MFI between 3,000 and 7,999, and 10 (14.5%) showed high-level MFI ≥8,000. Of these patient groups, there were no statistically significant differences in the characteristic of pre-sensitizing HLA between the shared TE-positive group (n=40) and shared TE-negative group (n=29), while the shared BE-positive status was seen more frequently in the shared TE-positive group (**Table 2**).

**Table 2:** Characteristics of non-DSA with the highest MFI.

Characteristics of Non-DSA with the highest MFI value	Anti-HLA ab (non-DSA)		*P*-value
	Shared TE-positive	Shared TE-negative	
	n = 40	n = 29	
**Non-DSA with the highest MFI value, n (%)**			0.27
HLA class I	23 (57.5)	21 (72.4)	
HLA class II	9 (22.5)	6 (20.7)	
HLA class I & II	8 (20)	2 (6.9)	
**The highest MFI value of non-DSA, n (%)**			0.66
1000–2999	20 (50)	17 (58.6)	
3000–7999	13 (32.5)	9 (31.0)	
8000–	7 (17.5)	3 (10.3)	
**Shared BE-positive, n (%)**	34 (85)	13 (44.8)	<0.001

Ab, antibody; non-DSA, non-donor-specific anti-HLA antibodies; TE, T cell epitope; MFI, mean fluorescence intensity; BE, B cell epitope.

### Characteristics of dnDSA

In this cohort, dnDSA were found in 52 of 578 patients (9.0%) during the full observational period, including HLA class I (n=5), DR (n=13), DQ (n=28), and DR+DQ (n=6). The median time to first detection was 26.5 months post-transplantation (IQR 11.75-37.5 months; range 0-84 months). Predominant dnDSA was directed against HLA class II (n=47), particularly DQ (n=34) and then DR (n=13). The incidence of class I DSA was low (n=5). The median highest MFI of dnDSA at the time of the first detection was 4,472.5 (IQR 2,070.5-1,1053.5) (**Table 3**).

**Table 3:** Characteristics of dnDSA in 578 patients without preformed DSA during the full observational period.

Characteristics of dnDSA	Anti-HLA ab (non-DSA)		No anti-HLA abn = 509	*P*-value
	Shared TE-positive	Shared TE-negative		
	n = 40	n = 29		
**dnDSA, n (%)**	8 (20)	0 (0)	44 (8.6)	0.58
HLA class I	1 (2.5)	0 (0)	4 (0.8)	
HLA class II	7 (17.5)	0 (0)	40 (7.9)	
HLA class I & II	0 (0)	0 (0)	0 (0)	
**The highest MFI value of dnDSA, n (%)**				0.15
1000–2999	3 (7.5)	–	18 (3.5)	
3000–7999	4 (10)	–	10 (2.0)	
8000–	1 (2.5)	–	16 (3.1)	

Ab, antibody; TE, T cell epitope; dnDSA, de novo donor-specific anti-HLA antibodies; MFI, mean fluorescence intensity.

### Impact of the Shared TEs on dnDSA Formation: Comparison With the Shared BEs

**Figure 3:**
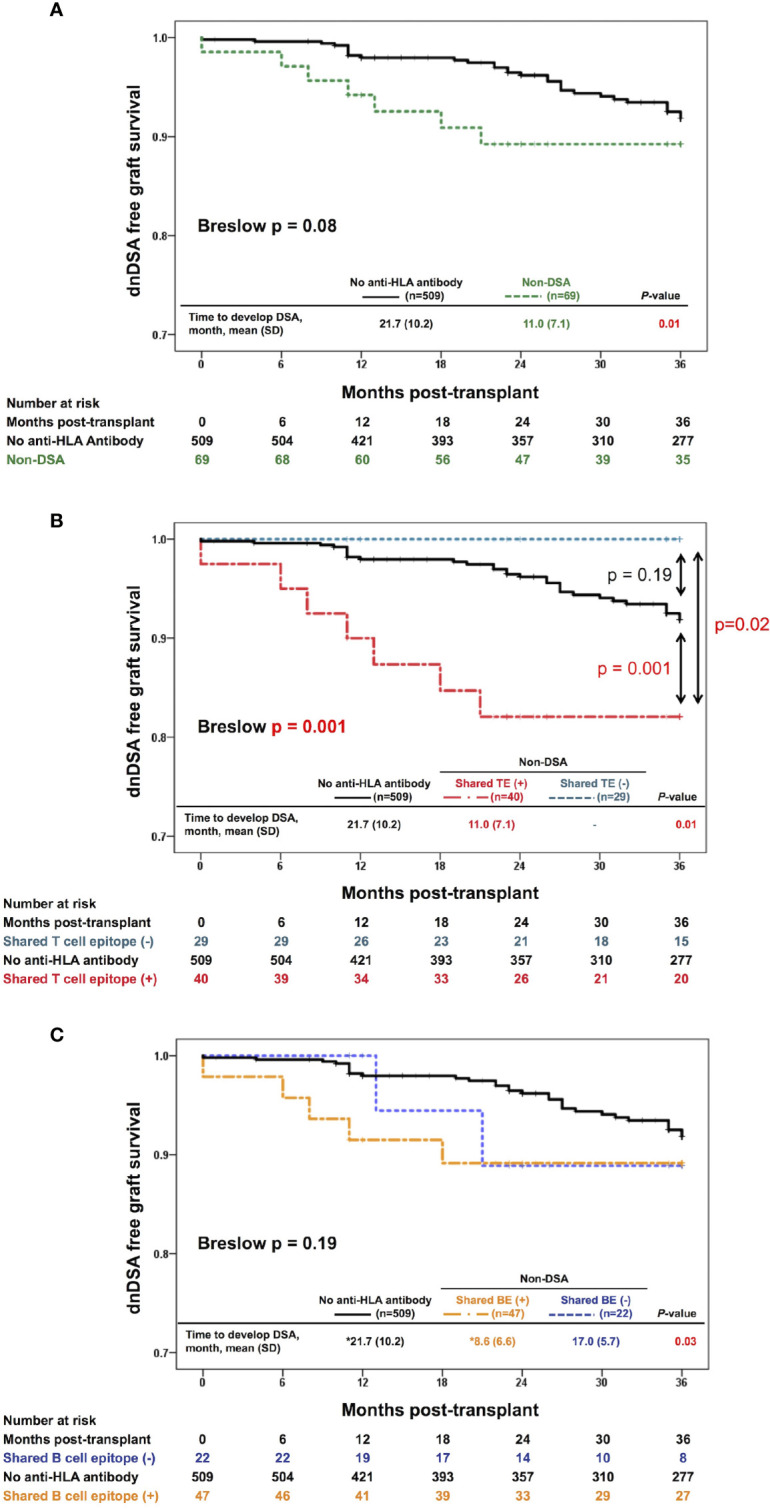
Effect of the shared TEs on dnDSA formation. **(A)** Three-year-dnDSA-free graft survival in the non-DSA versus no anti-HLA antibody group. The Kaplan-Meier curves and Breslow test tend to show higher incidences of dnDSA in the early phase after transplantation in the non-DSA group than in the no anti-HLA antibody group, although this trend is not statistically significant (p = 0.08). The time to develop DSA after transplantation is statistically earlier in the non-DSA group than in the no anti-HLA antibody group (p = 0.01). **(B)** Three-year-dnDSA-free graft survival in the three groups: shared TE-positive, shared TE-negative, and no anti-HLA antibody group. The Kaplan-Meier curves and Breslow test show a significant difference between these groups (p = 0.001). The shared TE-positive group shows significantly higher incidences of dnDSA than the no anti-HLA antibody group (p = 0.001), while there is no statistically significant difference between the shared TE-negative group and no anti-HLA antibody group (p = 0.19). The time to develop DSA after transplantation is statistically earlier in the shared TE-positive group than in the no anti-HLA antibody group (p = 0.01). **(C)** Three-year-dnDSA-free graft survival in three groups: shared BE-positive, shared BE-negative, and no anti-HLA antibody group. The Kaplan-Meier curves and Breslow test show no significant difference between these groups (p = 0.19). The time to develop DSA after transplantation is statistically different between these groups (p = 0.03). *Multiple comparison results show statistical differences between only the shared BE-positive and no anti-HLA antibody group. dnDSA, *de novo* donor-specific anti-HLA antibodies; non-DSA, non-donor-specific anti-HLA antibodies; TE, T cell epitope; BE, B cell epitope; SD, standard deviation.

To highlight the clinical impact of** **the pre-transplant memory CD4^+^ T cells rather than the primary naïve immune response, we focused on the 3-year observational period after transplantation, which is a relatively early phase in the overall follow-up period of this study (median, 47 months; IQR, 29-71.75 months; range, 1-95 months). Within 3 years after organ transplantation, 38 patients were diagnosed with positive dnDSA. In this period, the non-DSA group tended to show higher incidences of dnDSA during the early phase after transplantation than the no anti-HLA antibody group, although this trend was not statistically significant (p=0.08) (**Figure 3A**). The non-DSA group was then divided into the shared TE-positive and shared TE-negative groups. The shared TE-positive group showed significantly higher incidences of dnDSA than the shared TE-negative group (p=0.02) and no anti-HLA antibody group (p=0.001), while there was no statistically significant difference between the shared TE-negative group and no anti-HLA antibody group (p=0.19). The time to develop DSA after transplantation was statistically earlier in the shared TE-positive group than in the no anti-HLA antibody group (**Figure 3B**). We also checked the contributions of shared BEs on the development of dnDSA during the same period. Similar to the shared TE-positive group, the time to develop DSA after transplantation was statistically earlier in the shared BE-positive group than in the no anti-HLA antibody group; however, there were no statistically significant differences between the shared BE-positive and other groups in the analysis of DSA-free survival (**Figure 3C**).

### Analysis on the Association Between the TE and BE

**Figure 4:**
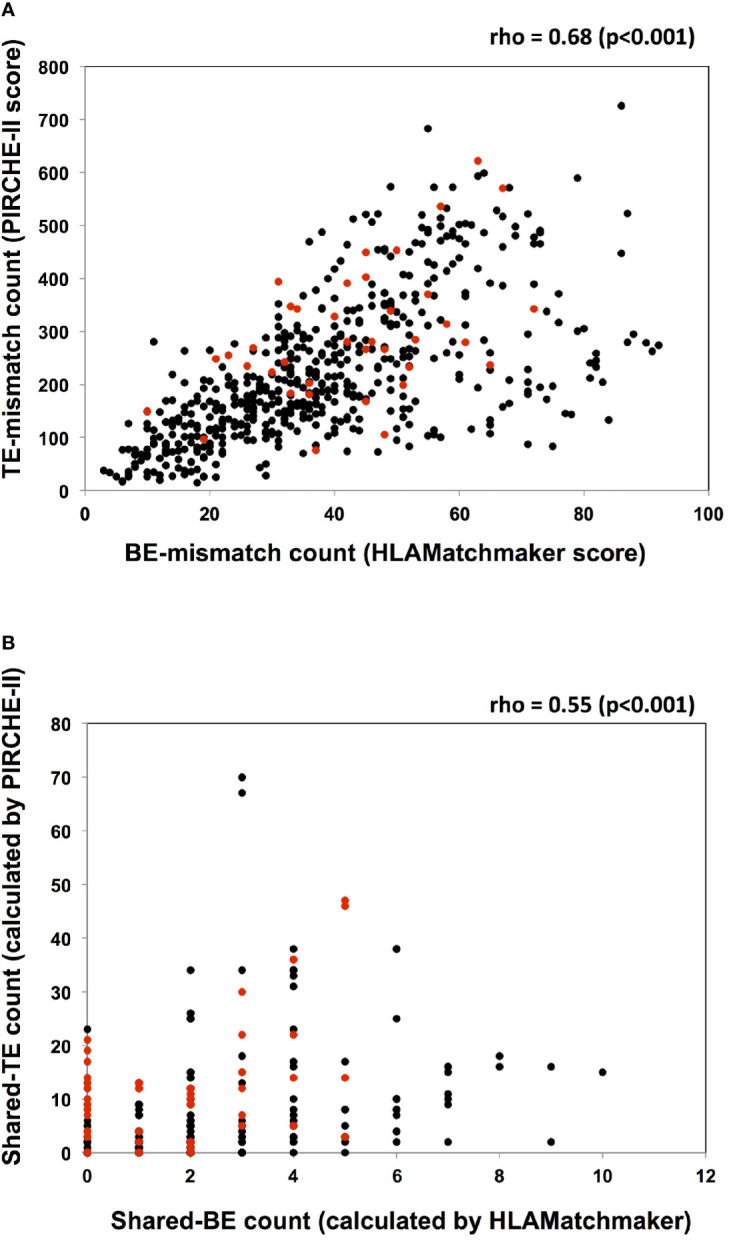
Analyses of the association between TE and BE. **(A)** The TE-mismatch count (PIRCHE-II score) and the BE-mismatch count (HLAMatchmaker score) of each recipient and donor pair are plotted. A moderately positive correlation between the TE- and BE-mismatch counts is noted with a Spearman’s rho of 0.68 (p < 0.001). Red circles and black circles indicate the dnDSA positive and negative patients, respectively. **(B)** The shared-TE count (calculated by PIRCHE-II) and the shared-BE count (calculated by HLAMatchmaker) between every detected non-DSA (MFI>1000) and the donor HLA are plotted. Each characteristic of non-DSA and the corresponding shared-TE and shared-BE counts are listed in **Supplementary Tables 1** and **2**. There is a weakly positive correlation between the shared-TE and shared-BE counts, with a Spearman’s rho of 0.55 (p < 0.001). Red circles and black circles indicate non-DSA in dnDSA positive and negative patients, respectively. dnDSA, *de novo* donor-specific anti-HLA antibodies; non-DSA, non-donor-specific anti-HLA antibodies; TE, T cell epitope; BE, B cell epitope; PIRCHE, predicted indirectly recognizable HLA epitopes.

There was a moderately positive correlation between the TE-mismatch count (PIRCHE-II score) and the BE-mismatch count (HLAMatchmaker score), with a Spearman’s rho of 0.68 (p < 0.001; **Figure 4A**). Conversely, the positive correlation between the shared-TE and shared-BE counts was weaker than that between the TE-mismatch and BE-mismatch counts (rho = 0.55, p<0.001) (**Figure 4B**). In each analysis, 3-year-dnDSA-positive patients (plotted in red) showed positive correlation to a lesser extent than all patients (TE- and BE-mismatch analysis; rho = 0.65, p<0.001 and shared-TE and -BE analysis; rho = 0.33, p = 0.006).

### Risk Factors Associated With dnDSA Formation

During the 3-year observational period, shared TE-positive status, and ln(PIRCHE-II) were associated with an increased risk of dnDSA development in univariate Cox proportional hazards regression modeling. Furthermore, shared TE-positive status and ln(PIRCHE-II) remained significant in multivariate analysis. The PIRCHE-II score of shared TE-positive patients (median, 184.5; IQR, 120–280.5) was slightly lower than that of the total patients (median, 196; IQR, 129–291), and the adjustment for this difference might result in increased hazard ratio of shared TE-positive status in multivariate analysis. Shared BE-positive status was not associated with dnDSA formation in univariate Cox proportional hazards regression models (**Table 4-1**). We also included a longer observation period that spanned 5 years after transplantation. Univariate Cox proportional hazards regression modeling revealed that during this period, the shared TE-positive status, ln(PIRCHE-II), and HLAMatchmaker score were associated with an increased risk of dnDSA development. Although a longer observation period and HLA locus-specific analysis improve the power of the HLAMatchmaker score as a risk predictor for dnDSA formation, the shared TE-positive status and ln(PIRCHE-II) also remained significant risk factors in multivariate analysis (**Table 4-2**).

**Table 4-1:** Cox proportional hazards models of factors associated with dnDSA production in 3 years (n = 38).

Variables	Univariate analysis		*P*-value	Multivariate analysis		*P*-value
	HR	95% CI		HR	95% CI	
ABO-I vs ABO-Id/C	0.83	0.42–1.65	0.60			
Anti-CD20 use	0.91	0.44–1.87	0.79			
Pre-sensitizing event	0.80	0.41–1.59	0.53			
Count of HLA mismatches^*^ per 1 increment	1.00	0.89–1.13	0.97			
Shared BE-positive	1.70	0.66–4.36	0.27			
Shared TE-positive^**^	3.37	1.48–7.65	0.004	3.80	1.66–8.67	0.002
ln(PIRCHE-II) score per 1 increment^**^	2.81	1.56–5.05	0.001	3.29	1.62–6.67	0.001
HLAMatchmaker score (A, B, DRB1/3/4/5, DQB1, and DQA1) per 10 increments	1.13	0.96–1.33	0.13			
HLAMatchmaker score (DRB1/3/4/5, DQB1, and DQA1) per 10 increments^**^	1.18	0.99–1.40	0.073	0.95	0.75–1.21	0.68

**Table 4-2:** Cox proportional hazards models of factors associated with dnDSA production in 5 years (n = 50).

Variables	Univariate analysis		*P*-value	Multivariate analysis		*P*-value
	HR	95% CI		HR	95% CI	
ABO-I vs ABO-Id/C	0.63	0.34–1.19	0.15			
Anti-CD20 use	0.67	0.33–1.34	0.25			
Pre-sensitizing event	0.73	0.40–1.33	0.30			
Count of HLA mismatches^*^ per 1 increment	1.02	0.92–1.13	0.66			
Shared BE-positive	1.54	0.66–3.61	0.32			
Shared TE-positive^**^	2.99	1.40–6.37	0.005	3.45	1.60–7.41	0.002
ln(PIRCHE-II) score per 1 increment^**^	2.71	1.64–4.50	<0.001	2.72	1.50–4.92	0.001
HLAMatchmaker score (A, B, DRB1/3/4/5, DQB1, and DQA1) per 10 increments	1.19	1.04–1.37	0.011			
HLAMatchmaker score (DRB1/3/4/5, DQB1, and DQA1) per 10 increments^**^	1.24	1.07–1.44	0.005	1.06	0.87–1.28	0.57

^*^HLA mismatch consists of mismatch at HLA-A, B, DRB1/3/4/5, DQB1, and DQA1 loci.

^**^A multivariate analysis with forced entry model was generated using the univariate factors. Only HLAMatchmaker score (DRB1/3/4/5, DQB1, and DQA1) per 10 increments, ln(PIRCHE-II) score per 1 increment, and shared TE-positive were included in the multivariate analysis.

dnDSA, de novo donor-specific anti-HLA antibody; HR, hazard ratio; CI, confidence interval; ABO-I, ABO-incompatible transplantation; ABO-Id/C, ABO-identical/compatible transplantation; non-DSA, non donor-specific anti-HLA antibody; BE, B cell epitope; TE, T cell epitope; PIRCHE, predicted indirectly recognizable HLA epitopes; ln (PIRCHE-II), natural logarithm of the PIRCHE-II scores.

### Cumulative Incidence of Adverse Outcomes in 5 Years

During the 5-year observational period after transplantation, cumulative dnDSA production occurred in 8 of 40 patients (20%) in the shared TE-positive group, in 0 of 29 patients (0%) in the shared TE-negative group, and in 42 of 509 (8.3%) patients in the no anti-HLA antibody group. The cumulative incidences of dnDSA were significantly higher in the shared TE-positive group compared with the other two groups during the full observational period; the onset appeared to be more frequent in the early phase, especially until 2 years after transplantation (p<0.001). Death-censored graft loss within 5 years after transplantation occurred in 3 (7.5%) patients in the shared TE-positive group, 0 (0%) patients in the shared TE-negative group, and 14 (2.8%) patients in the no anti-HLA antibody group (**Table 5**). There were no statistically significant differences in graft loss and rejection between these groups, although the number of the incidence of these events was low. Focusing on the 8 dnDSA cases in the shared TE-positive group, graft loss was observed in 2 patients, while antibody-mediated rejection was not observed within 5 years after transplantation; to evaluate the deleterious impact of dnDSA on the prognosis after transplantation, a longer observational period is required.

**Table 5:** Cumulative incidences of graft loss, rejection, and dnDSA.

Adverse outcomes	Anti-HLA ab (non-DSA)		No anti-HLA ab n = 509	*P*-value
	Shared TE-positive	Shared TE-negative		
	n = 40	n = 29		
**Functional, n(%)**				
5y graft loss	3 (7.5)	0 (0)	19 (3.7)	0.27
5y death censored graft loss	3 (7.5)	0 (0)	14 (2.8)	0.15
**Immunological, n (%)**				
5y antibody-mediated rejection	0 (0)	0 (0)	8 (1.6)	0.58
5y T cell-mediated rejection	2 (5)	1 (3.4)	29 (5.7)	0.87
5y dnDSA*	8 (20)	0 (0)	42 (8.3)	0.009
3y dnDSA*	7 (17.5)	0 (0)	31 (6.0)	0.007
2y dnDSA*	7 (17.5)	0 (0)	17 (3.3)	<0.001
1y dnDSA*	4 (10)	0 (0)	10 (2.0)	0.004

*Accumulating number of dnDSA-detected patients during each observational period, which include both persisting and disappearing dnDSA.

Ab, antibody; non-DSA, non-donor-specific anti-HLA antibodies; TE, T cell epitope; 5/3/2/1y, 5/3/2/1-year; dnDSA, de novo donor-specific anti-HLA antibodies.

### Characteristics of Shared pHLAs in the Shared TE-Positive Group

Last, we focused on the shared TE-positive group. The details of shared pHLAs in 3-year-dnDSA-positive cases (n=7) are shown in **Table 6**. In three cases (patient number 29, 38, and 40), detected dnDSA were directed to the same HLA as the origin of the shared peptide; however, in the remaining 4 cases, the origin of the shared peptide was not the same HLA as the target of dnDSA. In comparison to the 3-year dnDSA-positive versus dnDSA-negative group in the shared TE-positive group, shared pHLAs tended to be derived from only HLA class I in the 3-year dnDSA-negative group, although this trend was not statistically significant (**Table 7**).

**Table 6:** Details of shared pHLAs; 3-year dnDSA-positive cases in the shared TE-positive group (n = 7).

Pt	DnDSA		Presenting HLA loci	Presented shared peptide	The origin of shared peptide		
No.					Pre-sensitizing HLA	Donor HLA	
1	DQB1*06:04		DRB1*14:06	ITQRKWEAARVAEQL	B*54:01	A*33:03, B*44:03	
			DRB1*14:06	QRKWEAARVAEQLRA	B*54:01	A*33:03, B*44:03	
			DRB1*14:06	QLRAYLEGTCVEWLR	B*54:01	A*33:03	
			DRB1*14:06	RAYLEGTCVEWLRRY	B*54:01	A*33:03	
			DQA1*05:03 DQB1*03:01	QLRAYLEGTCVEWLR	B*54:01	A*33:03	
			DQA1*05:03 DQB1*03:01	AQITQRKWEAARVAE	B*54:01	A*33:03, B*44:03	
			DQA1*05:03 DQB1*03:01	QRKWEAARVAEQLRA	B*54:01	A*33:03, B*44:03	
5	DRB3*03:01,		DQA1*01:03 DQB1*06:01	AQITQRKWEAARVAE	B*37:01	A*33:03, B*44:03	
	DRB4*01:03						
7	DQB1*03:01		DRB1*13:02	SMRYFYTSVSRPGRG	A*26:01	A*02:06	
			DRB1*13:02	SHSMRYFYTSVSRPG	A*26:01	A*02:06	
			DRB1*13:02	HSMRYFYTSVSRPGR	A*26:01	A*02:06	
			DRB1*15:02	SMRYFYTSVSRPGRG	A*26:01	A*02:06	
			DRB1*15:02	SHSMRYFYTSVSRPG	A*26:01	A*02:06	
			DRB3*03:01	SHSMRYFYTSVSRPG	A*26:01	A*02:06	
			DRB3*03:01	MRYFYTSVSRPGRGE	A*26:01	A*02:06	
			DRB3*03:01	HSMRYFYTSVSRPGR	A*26:01	A*02:06	
			DRB5*01:02	AVVAAVMWRRKSSDR	A*26:01	A*02:06	
			DRB5*01:02	YTSVSRPGRGEPRFI	A*26:01	A*02:06	
			DRB5*01:02	KETLQRTDAPKTHMT	A*26:01	A*02:06	
			DRB5*01:02	SMRYFYTSVSRPGRG	A*26:01	A*02:06	
			DRB5*01:02	MRYFYTSVSRPGRGE	A*26:01	A*02:06	
			DQA1*01:02 DQB1*06:04	SHSMRYFYTSVSRPG	A*26:01	A*02:06	
			DQA1*01:03 DQB1*06:01	SHSMRYFYTSVSRPG	A*26:01	A*02:06	
			DQA1*01:03 DQB1*06:01	SMRYFYTSVSRPGRG	A*26:01	A*02:06	
			DQA1*01:03 DQB1*06:04	SHSMRYFYTSVSRPG	A*26:01	A*02:06	
8		DRB4*01:03	DRB1*01:01	DIVADHVASYGVNLY	DQA1*05:01	DQA1*03:02	
			DRB1*01:01	EDIVADHVASYGVNL	DQA1*05:01	DQA1*03:02	
			DRB1*01:01	FDPQFALTNIAVLKH	DQA1*05:01	DQA1*03:02	
			DRB1*01:01	ASYGVNLYQSYGPSG	DQA1*05:01	DQA1*03:02	
			DRB1*01:01	VVNITWLSNGHSVTE	DQA1*05:01	DQA1*03:02	
			DRB1*01:01	VADHVASYGVNLYQS	DQA1*05:01	DQA1*03:02	
			DRB1*15:02	QFALTNIAVLKHNLN	DQA1*05:01	DQA1*03:02	
			DRB1*15:02	PVVNITWLSNGHSVT	DQA1*05:01	DQA1*03:02	
			DRB1*15:02	FDPQFALTNIAVLKH	DQA1*05:01	DQA1*03:02	
			DRB1*15:02	PQFALTNIAVLKHNL	DQA1*05:01	DQA1*03:02	
			DRB1*15:02	ADHVASYGVNLYQSY	DQA1*05:01	DQA1*03:02	
			DRB5*01:02	QFALTNIAVLKHNLN	DQA1*05:01	DQA1*03:02	
			DRB5*01:02	VVNITWLSNGHSVTE	DQA1*05:01	DQA1*03:02	
			DQA1*01:01 DQB1*06:01	ITWLSNGHSVTEGVS	DQA1*05:01	DQA1*03:02	
			DQA1*01:01 DQB1*06:01	TWLSNGHSVTEGVSE	DQA1*05:01	DQA1*03:02	
			DQA1*01:01 DQB1*06:01	RSNSTAATNEVPEVT	DQA1*05:01	DQA1*03:02	
			DQA1*01:01 DQB1*06:01	ADHVASYGVNLYQSY	DQA1*05:01	DQA1*03:02	
			DQA1*01:01 DQB1*06:01	PQFALTNIAVLKHNL	DQA1*05:01	DQA1*03:02	
			DQA1*01:01 DQB1*06:01	KRSNSTAATNEVPEV	DQA1*05:01	DQA1*03:02	
			DQA1*01:01 DQB1*06:01	FDPQFALTNIAVLKH	DQA1*05:01	DQA1*03:02	
			DQA1*01:01 DQB1*06:01	QFALTNIAVLKHNLN	DQA1*05:01	DQA1*03:02	
			DQA1*01:01 DQB1*06:01	DIVADHVASYGVNLY	DQA1*05:01	DQA1*03:02	
			DQA1*01:03 DQB1*05:01	ADHVASYGVNLYQSY	DQA1*05:01	DQA1*03:02	
			DQA1*01:03 DQB1*05:01	VASYGVNLYQSYGPS	DQA1*05:01	DQA1*03:02	
			DQA1*01:03 DQB1*06:01	NITWLSNGHSVTEGV	DQA1*05:01	DQA1*03:02	
			DQA1*01:03 DQB1*06:01	IVADHVASYGVNLYQ	DQA1*05:01	DQA1*03:02	
			DQA1*01:03 DQB1*06:01	VNITWLSNGHSVTEG	DQA1*05:01	DQA1*03:02	
			DQA1*01:03 DQB1*06:01	TWLSNGHSVTEGVSE	DQA1*05:01	DQA1*03:02	
			DQA1*01:03 DQB1*06:01	ADHVASYGVNLYQSY	DQA1*05:01	DQA1*03:02	
			DQA1*01:03 DQB1*06:01	FDPQFALTNIAVLKH	DQA1*05:01	DQA1*03:02	
			DQA1*01:03 DQB1*06:01	PQFALTNIAVLKHNL	DQA1*05:01	DQA1*03:02	
			DQA1*01:03 DQB1*06:01	ITWLSNGHSVTEGVS	DQA1*05:01	DQA1*03:02	
			DQA1*01:03 DQB1*06:01	WLSNGHSVTEGVSET	DQA1*05:01	DQA1*03:02	
			DQA1*01:03 DQB1*06:01	DIVADHVASYGVNLY	DQA1*05:01	DQA1*03:02	
29		A*01:01	DQA1*03:02 DQB1*03:03	ALNEDLRSWTAADMA	A*34:01	A*01:01	
			DQA1*05:03 DQB1*03:03	IALNEDLRSWTAADM	A*34:01	A*01:01	
			DRB1*09:01	DTYCRHNYGVVESFT	DRB1*15:01	DRB1*04:03	
			DQA1*03:02 DQB1*03:01	YCRHNYGVVESFTVQ	DRB1*15:01	DRB1*04:03	
			DQA1*05:03 DQB1*03:01	YCRHNYGVVESFTVQ	DRB1*15:01	DRB1*04:03	
			DQA1*05:03 DQB1*03:03	YCRHNYGVVESFTVQ	DRB1*15:01	DRB1*04:03	
38		DRB1*04:01	DRB1*15:01	EVTVYPAKTQPLQHH	DRB1*09:01	DRB1*04:01	
			DRB5*01:01	EVTVYPAKTQPLQHH	DRB1*09:01	DRB1*04:01	
			DQA1*01:02 DQB1*06:02	RNGQEEKAGVVSTGL	DRB1*09:01	DRB4*01:02	
40		DRB5*01:01,	DRB1*08:03	RFDPQFALTNIAVLK	DQA1*05:05	DQA1*03:03
		DQA1*03:03	DRB1*08:03	HVASYGVNLYQSYGP	DQA1*05:05	DQA1*03:03
			DRB1*08:03	NITWLSNGHSVTEGV	DQA1*05:05	DQA1*03:03
			DRB1*08:03	DIVADHVASYGVNLY	DQA1*05:05	DQA1*03:03
			DRB1*08:03	IKRSNSTAATNEVPE	DQA1*05:05	DQA1*03:03
			DRB1*08:03	QFALTNIAVLKHNLN	DQA1*05:05	DQA1*03:03
			DRB1*08:03	PVVNITWLSNGHSVT	DQA1*05:05	DQA1*03:03
			DRB1*08:03	ADHVASYGVNLYQSY	DQA1*05:05	DQA1*03:03
			DRB1*13:02	VVNITWLSNGHSVTE	DQA1*05:05	DQA1*03:03
			DRB1*13:02	EDIVADHVASYGVNL	DQA1*05:05	DQA1*03:03
			DRB1*13:02	QFALTNIAVLKHNLN	DQA1*05:05	DQA1*03:03
			DRB3*03:01	QFALTNIAVLKHNLN	DQA1*05:05	DQA1*03:03
			DRB3*03:01	RFDPQFALTNIAVLK	DQA1*05:05	DQA1*03:03
			DRB3*03:01	PVVNITWLSNGHSVT	DQA1*05:05	DQA1*03:03
			DRB3*03:01	EDIVADHVASYGVNL	DQA1*05:05	DQA1*03:03
			DRB3*03:01	ADHVASYGVNLYQSY	DQA1*05:05	DQA1*03:03
			DRB3*03:01	VASYGVNLYQSYGPS	DQA1*05:05	DQA1*03:03
			DQA1*01:03 DQB1*06:01	NITWLSNGHSVTEGV	DQA1*05:05	DQA1*03:03
			DQA1*01:03 DQB1*06:01	IVADHVASYGVNLYQ	DQA1*05:05	DQA1*03:03
			DQA1*01:03 DQB1*06:01	VNITWLSNGHSVTEG	DQA1*05:05	DQA1*03:03
			DQA1*01:03 DQB1*06:01	TWLSNGHSVTEGVSE	DQA1*05:05	DQA1*03:03
			DQA1*01:03 DQB1*06:01	ADHVASYGVNLYQSY	DQA1*05:05	DQA1*03:03
			DQA1*01:03 DQB1*06:01	FDPQFALTNIAVLKH	DQA1*05:05	DQA1*03:03
			DQA1*01:03 DQB1*06:01	PQFALTNIAVLKHNL	DQA1*05:05	DQA1*03:03
			DQA1*01:03 DQB1*06:01	ITWLSNGHSVTEGVS	DQA1*05:05	DQA1*03:03
			DQA1*01:03 DQB1*06:01	WLSNGHSVTEGVSET	DQA1*05:05	DQA1*03:03
			DQA1*01:03 DQB1*06:01	DIVADHVASYGVNLY	DQA1*05:05	DQA1*03:03
			DQA1*01:03 DQB1*06:04	IVADHVASYGVNLYQ	DQA1*05:05	DQA1*03:03
			DQA1*01:03 DQB1*06:04	EDIVADHVASYGVNL	DQA1*05:05	DQA1*03:03
			DQA1*01:03 DQB1*06:04	ADHVASYGVNLYQSY	DQA1*05:05	DQA1*03:03
			DQA1*01:02 DQB1*06:01	PVVNITWLSNGHSVT	DQA1*05:05	DQA1*03:03
			DQA1*01:02 DQB1*06:01	NITWLSNGHSVTEGV	DQA1*05:05	DQA1*03:03
			DQA1*01:02 DQB1*06:01	IVADHVASYGVNLYQ	DQA1*05:05	DQA1*03:03
			DQA1*01:02 DQB1*06:01	RSNSTAATNEVPEVT	DQA1*05:05	DQA1*03:03
			DQA1*01:02 DQB1*06:01	DPQFALTNIAVLKHN	DQA1*05:05	DQA1*03:03
			DQA1*01:02 DQB1*06:01	ITWLSNGHSVTEGVS	DQA1*05:05	DQA1*03:03
			DQA1*01:02 DQB1*06:01	ADHVASYGVNLYQSY	DQA1*05:05	DQA1*03:03
			DQA1*01:02 DQB1*06:01	KRSNSTAATNEVPEV	DQA1*05:05	DQA1*03:03
			DQA1*01:02 DQB1*06:01	FDPQFALTNIAVLKH	DQA1*05:05	DQA1*03:03
			DQA1*01:02 DQB1*06:01	PQFALTNIAVLKHNL	DQA1*05:05	DQA1*03:03
			DQA1*01:02 DQB1*06:01	WLSNGHSVTEGVSET	DQA1*05:05	DQA1*03:03
			DQA1*01:02 DQB1*06:01	DIVADHVASYGVNLY	DQA1*05:05	DQA1*03:03
			DQA1*01:02 DQB1*06:04	FDPQFALTNIAVLKH	DQA1*05:05	DQA1*03:03
			DQA1*01:02 DQB1*06:04	IVADHVASYGVNLYQ	DQA1*05:05	DQA1*03:03
			DQA1*01:02 DQB1*06:04	EDIVADHVASYGVNL	DQA1*05:05	DQA1*03:03
			DQA1*01:02 DQB1*06:04	ADHVASYGVNLYQSY	DQA1*05:05	DQA1*03:03

dnDSA, de novo donor-specific anti-HLA antibodies; Pt, patient; pHLA, peptide in the context of recipient HLA class II; No., number; A, alanine; R, arginine; N, asparagine; D, aspartic acid; C, cysteine; Q, glutamine; E, glutamic acid; G, glycine; H, histidine; I, Isoleucine; L, leucine; K, lysine; M, methionine; F, phenylalanine; P, proline; S, serine; T, threonine; W, Tryptophan; Y, tyrosine; V, valine.

**Table 7:** Characteristics of shared pHLAs; comparison of 3-year dnDSA-positive versus negative group in the shared TE-positive group.

	3y dnDSA-positive	3y dnDSA-negative	*P*-value
	n = 7	n = 33	
**Total shared pHLAs count, median (IQR)**	7 (3–34)	4 (2–13)	0.17
**The origin of the shared pHLAs, n (%)**			
** Pre-sensitizing HLA**			0.39
HLA class I	3 (42.9)	23 (69.7)	
HLA class II	3 (42.9)	7 (21.2)	
HLA class I & II	1 (14.3)	3 (9.1)	
** Donor HLA**			0.44
HLA class I	3 (42.9)	22 (66.7)	
HLA class II	3 (42.9)	7 (21.2)	
HLA class I & II	1 (14.3)	4 (12.1)	

dnDSA, de novo donor-specific anti-HLA antibodies; 3y, 3-year; TE, T cell epitope; pHLA, peptide in the context of recipient HLA class II; IQR, interquartile range.

## Discussion

This study was the first attempt to use the PIRCHE-II algorithm as a tool to estimate preformed memory CD4^+^ T cells, which may be reactivated by encountering pHLAs derived from the donor HLA *via* the indirect allorecognition pathway. As the first step in this study, pre-sensitizing HLA before transplantation had to be determined in order to assess shared TEs between the pre-sensitizing HLA and donor HLA. Clinically, it is difficult to determine this pre-sensitizing HLA by only considering a patient’s medical history; thus, we focused on the characteristics of non-DSA as an objective tool to determine pre-sensitizing HLA. Anti-HLA antibodies toward a shared BE were reportedly diluted across multiple beads in a SAB assay, resulting in lowering the MFI values compared with antibodies toward a private epitope specific to a single HLA (35). Therefore, we considered that non-DSA with the highest MFI value may include antibodies toward private epitopes specific to pre-sensitizing HLA. As shown in **Supplementary Table 1**, the presence or absence of shared TEs between the donor HLA and non-DSA with the highest MFI value tended to represent the presence of shared TEs between the donor HLA and non-DSA with following ranks. In most cases in the shared TE-positive group (n=39/40), the non-DSA with the highest MFI, as well as the non-DSA with following ranks, shared TEs with the donor HLA. Furthermore, in the majority of the cases in the shared TE-negative group (n=23/29), both the non-DSA with the highest MFI and the non-DSA with following ranks did not share TEs with the donor HLA. These findings suggest that the use of non-DSA with the highest MFI as a predictor for pre-sensitizing HLA might be effective to some extent; however, we acknowledge that more cases are required for validation. Additionally, we analyzed the effect of shared BEs between the pre-sensitizing HLA and donor HLA on post-transplantation outcomes because the non-DSA toward BEs shared with the donor HLA were conventionally believed to be an immunological risk (15). In this study, 69 cases with non-DSA were analyzed and 47 cases were determined to share BEs between the pre-sensitizing HLA and donor HLA. As shown in **Supplementary Table 2**, the results for shared TEs did not always match the results for shared BEs. Some cases from the shared TE-positive group were determined to be shared BE-negative (n=6/40), while some cases from the shared TE-negative group were determined to be shared BE-positive (n=13/29). A previous report has suggested a moderately positive correlation between the TE-mismatch count (PIRCHE-II score) and the BE-mismatch count (HLAMatchmaker score) (29); in our study as well, a moderately positive correlation between the TE- and BE-mismatch counts was noted (**Figure 4A** **)**. However, such a correlation was weakened between the shared-TE and shared-BE counts when the analysis was focused on the epitope shared between the non-DSA and donor HLA (**Figure 4B** **)**. This weak correlation may cause 69 cases with non-DSA to be stratified differently between shared TE and BE, and lead to different outcomes on early dnDSA formation. In this cohort, the time to develop DSA after transplantation was statistically earlier in the shared BE-positive group than in the no anti-HLA antibody group; however we could not ascertain that shared BEs are a significant risk factor for early dnDSA formation (**Figure 3C**; **Tables 4-1** and **4-2**). Instead, shared TEs between the pre-sensitizing HLA and donor HLA were suggested to be a significant risk of dnDSA formation, especially in the early phase, implying the contribution of preformed donor-reactive memory CD4^+^ T cells rather than the primary naïve immune response (**Figure 3B**). Additionally, in the multivariable Cox proportional hazards models, the shared TEs were suggested to be an independent risk factor affecting early dnDSA formation together with ln(PIRCHE-II), which was previously reported to be a risk factor (29, 30) (**Table 4**). Importantly, in our study, the non-DSA group tended to show higher incidences of dnDSA in the early phase after transplantation than the no anti-HLA antibody group, although this trend was not statistically significant (p=0.08) (**Figure 3A** **)**; it remains controversial whether or not preformed non-DSA and their varieties could be an immunological risk for impaired graft survival (36, 37). Our method clearly stratified non-DSA into deleterious or not deleterious by analyzing shared TEs. Additionally focusing on the shared TEs, PIRCHE-estimated-APC-presented peptides were not always derived from the same HLA as the target of dnDSA in the shared TE-positive group (**Table 6**). Similar to the shared TEs, HLAMatchmaker-estimated-shared BEs were not always derived from the same HLA as the target of dnDSA. For example, in one patient (shared-TE (+) - 5, in **Supplementary Table 2**), the shared TEs and BEs between the non-DSA and donor HLA were all derived from HLA class I, while the target of the detected dnDSA was HLA class II. This finding implied that the HLAs boosting memory CD4^+^ T cells could be different from the HLAs that were targeted by antibodies. This could occur when patients were exposed to multiple HLAs during organ transplantation. These results supported our hypothesis that preformed donor-reactive memory CD4^+^ T-helper cells activated by the shared TEs play a crucial role in promoting early dnDSA formation, even though there are no preformed DSA and their associated plasma cells and memory B cells.

However, we acknowledge that our methods may be a pseudomarker for T cell-responses, and this method has several limitations. First, this retrospective study in a single center features a relatively small sample size and brief observational period. Second, almost half of the patients with preformed non-DSA did not have any known pre-sensitizing event, which might be the result of an unrecognized pre-sensitizing event, such as early miscarriages in women or heterologous immunity (38); furthermore, it might be the result of false-positive SAB analysis (37, 39). Third, non-DSA with the highest MFI value may not always reflect actual pre-sensitizing history because most of the cases have been sensitized by multiple HLAs in repeated pre-sensitizing event. Furthermore, MFI values of SAB assays have analytic limitations in terms of quantitativeness of the antibody amount (35). There is still room for improving the *in silico* analysis to determine actual pre-sensitizing HLAs. Fourth, although it was reported that anti-HLA-C/-DP sensitization was also deleterious in kidney transplantation, HLA-C and DP were not typed and taken into account for the definition of DSA in this study (40). Fifth, this study lacks high-resolution HLA genotyping data on DRB3/4/5 and DQA1; these missing data were extrapolated to second field HLA typing using a local haplotype frequency dataset of 916 unrelated Japanese individuals (23). While we acknowledge that recent reports suggest insufficient accuracy of imputed HLA alleles, especially in ethnically heterogeneous non-Caucasian individuals (41), single ethnicity of our patients in this study would lower such the error rate. Sixth, we could only assess preformed donor-reactive memory in the non-DSA-positive population, since we did not have objective evidence except for anti-HLA antibodies. Detection of preformed donor-reactive memory in the no anti-HLA antibody group would be the next target. Considering these limitations, the validity of our findings needs to be confirmed by combining them with *in vitro* assays.

Although previous studies suggest that the standard *in vitro* assay of detecting preformed donor-reactive memory T cells was interferon gamma ELISPOT assay (IFNγ ELISPOT) (42), IFNγ ELISPOT can detect such T cells dominantly activated *via* the direct allorecognition pathway (43). In fact, pre-transplantation IFNγ ELISPOT positivity is broadly reported to be related to a high risk of rejection in the early phase (18, 42, 44). The *in silico* assay used in our study was especially focused on detecting preformed donor-reactive memory T cells activated *via* the indirect allorecognition pathway; our results showed that this method was related to a high risk of early dnDSA formation (**Figure 3**). In terms of the clinical effect on each T cell allorecognition pathway, these results are quite reasonable (45); however, validation *via in vitro* assays with a focus on the indirect allorecognition pathway is still required.

In addition to pre-transplantation risk stratification, further therapeutic consideration will be needed to reduce risk and improve prognosis, especially with the limited supply of organs. A previous report suggested that anti-thymocyte globulin (ATG) has the potential to control donor-reactive memory T cells detected by IFNγ ELISPOT (42). Although further clinical trial is required, ATG could be a beneficial intervention, even in patients with donor-reactive memory T cells, which would be activated *via* the indirect allorecognition pathway.

In conclusion, the evaluation of shared TEs using the PIRCHE-II algorithm for the purpose of estimating preformed donor-reactive memory CD4^+^ T cells may help to predict the risk of early dnDSA formation after transplantation. Focusing on the pathogenesis of dnDSA formation, analysis of shared TEs is crucial for the precise understanding of the immune response to the donor organ, and should be distinguished from the conventional analysis of shared BEs. It remains difficult for *in vitro* assays to detect donor-reactive memory CD4^+^ T cells activated *via* the indirect allorecognition pathway. Our study suggests that the *in silico* assay using the PIRCHE-II algorithm may be an effective and alternative solution for estimating this pathway. Considering the various limitations in this study, a larger sample size and further clinical and basic scientific approaches will be needed to validate this emerging *in silico* assay.

## Data Availability Statement

The original contributions presented in the study are included in the article/**Supplementary Material**. Further inquiries can be directed to the corresponding author.

## Ethics Statement

The studies involving human participants were reviewed and approved by the Institutional Ethics Committees of Aichi Medical University Hospital and the Institutional Review Board of Nagoya Daini Red Cross Hospital. The patients/participants provided their written informed consent to participate in this study.

## Author Contributions

TT, KI, and TK designed the research. TT wrote the manuscript. TT, SS, KF, MO, TH, AT, NG, SN, YW, and TK performed the research. TT, MN, ES, and TK participated in data analysis. IN reviewed statistics. MN, ES, and TK reviewed the manuscript. All authors contributed to the article and approved the submitted version.

## Funding

This work was supported by JSPS KAKENHI Grant Numbers 20H03818 and 20K20608.

## Conflict of Interest

MN is an employee of PIRCHE AG. The UMC Utrecht has filed a patent application on the prediction of an alloimmune response against mismatched HLA. ES is listed as inventor on this patent.

The remaining authors declare that the research was conducted in the absence of any commercial or financial relationships that could be construed as a potential conflict of interest.
