# Erratum: Clinical significance of shared T cell epitope analysis in early *de novo* donor-specific anti-HLA antibody production after kidney transplantation and comparison with shared B cell epitope analysis

**DOI:** 10.3389/fimmu.2022.995022

**Published:** 2022-08-03

**Authors:** Frontiers Production Office

**Affiliations:** Frontiers Media SA, Lausanne, Switzerland

**Keywords:** kidney transplantation, T cell epitope analysis, B cell epitope analysis, donor-specific antibody, PIRCHE-II

Due to a production error, numbered headings were removed from this article but two citations of a numbered heading remained.

A correction has been made to the section **Materials and Methods**, subsection **Shared TE-Analysis as a Tool to Estimate Donor-Reactive Memory CD4+ T-Helper Cells**, Paragraph 2:

“In patients with preformed non-DSA, TEs derived from presensitizing HLA were calculated using PIRCHE-II and compared with calculated TEs derived from the donor HLA. We considered the shared TEs to be positive and estimated the presence of donor-reactive memory CD4+ T-helper cells if the two sets of TEs shared at least one pHLA, and negative if no pHLA were shared (Figure 1); for each non-DSA, TE counts shared with the donor HLA are shown in Supplementary Table 1.”

A correction has been made to the section **Materials and Methods**, subsection **Shared BE Analysis**, Paragraph 1:

“In patients with preformed non-DSA, similarly to the shared-TE analysis, the BEs derived from pre-sensitizing HLA were estimated using the HLAMatchmaker software and were compared with the calculated BEs derived from the donor HLAs. The shared BEs were determined as positive if the two sets of BEs shared at least one eplet, and negative if no eplets were shared; for each non-DSA, the BE counts shared with the donor HLA are shown in Supplementary Table 2.”

The publisher apologizes for this mistake. The original version of this article has been updated.

